# Prevention of ventilator-associated pneumonia in intensive care units: an international online survey

**DOI:** 10.1186/2047-2994-2-9

**Published:** 2013-03-26

**Authors:** Marie-Laurence Lambert, Mercedes Palomar, Antonella Agodi, Michael Hiesmayr, Alain Lepape, Anne Ingenbleek, Eduardo Palencia Herrejon, Stijn Blot, Uwe Frank

**Affiliations:** 1 Healthcare associated infections unit, Scientific Institute of Public Health, Rue Juliette Wytsmanstraat 14, Brussels, 1050, Belgium; 2 Intensive care department, Hospital Vall d’Hebron, Barcelona, Spain; 3 Universitat Autonoma de Barcelona, Barcelona, Spain; 4 Department GF Ingrassia, University of Catania, Catania, Italy; 5 Department Anaesthesia, General Intensive Care and Pain Control, Medical University of Vienna, Vienna, Austria; 6 Department Anaesthesia, General Intensive Care, University hospital, Lyon, France; 7 Intensive Care department, Hospital Universitario “Infanta Leonor”, Madrid, Spain; 8 Department of Internal Medicine, Ghent University, De Pintelaan 185, Ghent, 9000, Belgium; 9 Infectious diseases department, University hospital, Heidelberg, Germany

**Keywords:** Healthcare associated infection, Ventilator-associated pneumonia, Patient safety, Preventive measures, Quality of care

## Abstract

**Background:**

On average 7% of patients admitted to intensive-care units (ICUs) suffer from a potentially preventable ventilator-associated pneumonia (VAP). Our objective was to survey attitudes and practices of ICUs doctors in the field of VAP prevention.

**Methods:**

A questionnaire was made available online in 6 languages from April, 1^st^ to September 1^st^, 2012 and disseminated through international and national ICU societies. We investigated reported practices as regards (1) established clinical guidelines for VAP prevention, and (2) measurement of process and outcomes, under the assumption “if you cannot measure it, you cannot improve it”; as well as attitudes towards the implementation of a measurement system. Weighted estimations for Europe were computed based on countries for which at least 10 completed replies were available, using total country population as a weight. Data from other countries were pooled together. Detailed country-specific results are presented in an online additional file.

**Results:**

A total of 1730 replies were received from 77 countries; 1281 from 16 countries were used to compute weighted European estimates, as follows: care for intubated patients, combined with a measure of compliance to this guideline at least once a year, was reported by 57% of the respondents (95% CI: 54–60) for hand hygiene, 28% (95% CI: 24–33) for systematic daily interruption of sedation and weaning protocol, and 27% (95%: 23–30) for oral care with chlorhexidine. Only 20% (95% CI: 17–22) were able to provide an estimation of outcome data (VAP rate) in their ICU, still 93% (95% CI: 91–94) agreed that “Monitoring of VAP-related measures stimulates quality improvement”. Results for 449 respondents from 61 countries not included in the European estimates are broadly comparable.

**Conclusions:**

This study shows a low compliance with VAP prevention practices, as reported by ICU doctors in Europe and elsewhere, and identifies priorities for improvement.

## Introduction

Ventilator-associated pneumonia (VAP) is a frequent and severe health-care associated infection. In Europe, pneumonia occur in 7.0% of patients staying at least 2 days in intensive care units (ICUs); 91% of these pneumonia are VAP [1]. The proportion of VAP which is preventable is debated [2] but there is no doubt that a serious potential for harm reduction does exist [3,4] and VAP prevention is becoming a major patient safety issue. In the US for instance, VAP prevention has been proposed as a national safety goal [2].

VAP prevention requires clinical interventions (best practice guidelines) combined with non clinical interventions to ensure implementation and compliance with these guidelines.

Clinical interventions for VAP prevention fall in three categories [5]. The first, obvious one, is to limit exposure to mechanical ventilation by preferring non-mechanical ventilation when possible and limiting its duration when alternative options are not possible. Other prevention practices aim at reducing airways colonization (such as oral care decontamination using chlorhexidine [6]), or preventing aspiration [5] (e.g. by nursing in the semi-recumbent position, or maintaining a sufficient cuff pressure). Clinical interventions should be combined, in what is often called a “care bundle”. The precise content of VAP care bundle varies between guidelines [7,8] because the number of items in a “bundle” should be limited, some prevention practices are controversial (e.g. selective digestive decontamination), [9] and recommending best practice regarding such measures requires compromise and pragmatism [8]. There is no universally accepted “VAP care bundle”. A study aiming at defining a “European care bundle” ranked VAP prevention measures by combining criteria such as the strength of the supporting evidence, ease of implementation, and expected impact [10]. The top 5 clinical interventions (we did not consider “trained staff” as a clinical intervention) were: 1) no ventilatory circuit change unless specifically indicated; 2) strict hand hygiene with alcohol especially before managing the airways; 3) daily sedation vacation and weaning protocol; 4) oral care with chlorhexidine; and 5) cuff pressure control at least every 24 hrs. It was beyond the scope of our study to assess the evidence behind each of these interventions, but none appears to be controversial.

Ensuring compliance with guidelines is a vast and complex field of research [11-13]. Common to any improvement strategy is the need for measurement; this serves evaluation purposes, measurement can also be the intervention, or a major component of it [14-16]. In a survey on infection control practices in the US, ICUs were only able to reduce heathcare-associated infection rates (including VAP), when they had a written policy, monitored compliance, and achieved a ≥95% compliance to all elements included in the local care bundle [11,17]. Under the unchallenged assumption “if you cannot measure it, you cannot improve it” (Lord Kelvin, 1824-1907), we considered here that monitoring process (compliance to guidelines) and outcomes is a necessary, if not sufficient, component of any intervention aiming at decreasing VAP.

Accurate diagnosis of VAP is a challenge, because many conditions commonly encountered in critically ill patients – such as pulmonary oedema, pulmonary hemorrhage and acute respiratory distress syndrome, can mimic the signs and symptoms of pneumonia [5]. Clinical diagnosis leads to treatment decisions, its primary aim is to be accurate, and it cannot be entirely standardized. By contrast measurement need primarily to be reproducible to ensure comparability of data overtime and allow evaluation of trends. Standardization is essential. Criteria for diagnosis and criteria for recording, therefore do not necessarily overlap entirely. Guidelines and definitions of VAP for recording and reporting exist in Europe [18] and in the United States [19]. Despite some changes in the US, current case-definitions are still considered useful for internal quality improvement purposes [20].

Our objectives were to document, using a web-based survey (1) reported VAP prevention practices in ICUs (clinical practices, and measurement) and (2) attitudes towards the implementation of a measurement system. Our primary interest laid in providing estimates at European level, but we did not define exclusion criteria based on geographical location; on the other hand country-specific results can be used to steer prevention initiatives at country level.

## Methods

### Study population

Our target group was physicians working in ICUs. An ICU was defined as an unit meeting all the following criteria: provides facilities for invasive mechanical ventilation, and pump-controlled administration of infusion, functions 24 hours a day and 7 days a week, and there is at least one doctor immediately available at all times to deal with emergencies.

### Questionnaire

We developed a questionnaire with 3 parts: 1) Characteristics of the respondent and her/his ICU, 2) VAP prevention practices (clinical, and measurement). For clinical practices we focused on the top 5 clinical components of the European VAP care bundle [10] and added a commonly recommended practice (head of bed elevation) [5]. For measurement, we included questions on measurement of process (compliance to prevention practices, average duration of intubation) and outcomes (measurement of VAP, definitions used for data collection – European, [18] or American, [19] and the ability to report selected indicators. 3) attitudes as regards the implementation of a data collection system, using a 5 point-Likert scale (1: strongly agree, 5: strongly disagree) [13,21]. The questionnaire was kept very short to improve participation. It was first developed and pre-tested in English, then translated into German, Italian, Spanish, Portuguese and French by intensive care doctors and/or infection control practitioners, native speakers in the targeted language. Each translation had to be independently checked by at least another native speaker doctor. We used Limesurvey 2.0, an open source web survey application, to collect the data [22]. Participation was anonymous.

### Dissemination to target group

The questionnaire was available online from April 1 to September 1, 2012. It was endorsed by the European Society of Intensive Care Medicine (ESICM), posted on their website, and the link e-mailed to all its members. We contacted national ESICM representatives and key opinion leaders and requested their support in disseminating the survey in their country. The survey was endorsed by national ICU societies in Austria, France, Belgium, the Netherlands, Italy, and Greece. It was e-mailed to all subscribers of REMI (Revista Electronica de Medicina Intensiva), [23] an electronic newsletter on intensive care medicine in Spanish distributed in Spain, Portugal, and Latin America.

### Data analysis

Descriptive statistics were used to characterize the study sample. Using total country population (2012 United Nations estimates [24]) as the weight, we computed weighted European estimates including all countries which provided at least 10 completed replies. This arbitrary threshold was chosen as a compromise between the number of countries included in the European estimates, and precision of the estimation. Statistical software STATA 10 was used for the analyses *(svy* command for survey data for weighted estimates). Replies from the remaining countries, both European and non European, were simply pooled together. We choose to present uncommented, detailed country-specific results as Additional file 1, for use by national stakeholders.

## Results

A total of 1730 completed replies from 77 different countries were submitted (Table 1). Characteristics of the respondents, and their setting, are presented in Table 2.

**Table 1:** Number of replies to the survey, by country

	**Replies**	**%**
***European countries***		
Spain	293	17%
France	251	15%
Italy	187	11%
Austria	130	8%
United Kingdom	115	7%
Germany	67	4%
Portugal	50	3%
Belgium	33	2%
Netherlands	31	2%
Switzerland	29	2%
Greece	23	1%
Romania	20	1%
Denmark	15	1%
Sweden	14	1%
Ireland	13	1%
Hungary	10	1%
Total European countries with at least 10 replies*	*1281*	*74%*
Other (18 different countries)	55	3%
***Non European countries***		
India	63	4%
Argentina	40	2%
Colombia	31	2%
Mexico	31	2%
Australia	23	1%
Peru	23	1%
Brazil	21	1%
Ecuador	13	1%
Chile	12	1%
Turkey**	12	1%
United States	12	1%
Saudi Arabia	11	1%
United Arab Emirates	11	1%
Venezuela	11	1%
Other (29 different countries)	80	5%
**Survey - total**	1730	100%

* Used for European weighted estimates.

** Considered as non European as majority of population does not live in Europe.

**Table 2:** Characteristics of the respondents, and of their setting

**Respondent**	**Weighted estimates for Europe (respondents from 16 countries with >=10 replies)**			**Other respondents (61 countries)**			
	**N=1281**	**95% CI**		**N=449**		**95% CI**	
Years working in ICU (mean)	12.8	12.2	13.3	12.9		12.1	13.6
Admissions per year in their ICU (mean)	1006	914	1098	900		787	1013
N beds in ICU (mean)	16	15	17	16		15.4	17.3
	%*			*N*	*%*		
Gender (females)	28	25	31	82	18	15	22
Working in hospital with > 1000 beds	17	14	20	18	4	2	6
Working in hospital with 300–1000 beds	55	52	56	145	33	28	37
Working in hospital <300 beds	28	26	31	282	63	58	67

* Absolute numbers are not reported because percentages are weighted estimates.

Weighted European estimates are based on 1281 respondents from the 16 countries from which at least 10 completed replies were available.

VAP prevention practices are presented in Table 3 (clinical practices) and Table 4 (measurements).

**Table 3:** VAP prevention: clinical practices, as reported by ICU doctors

**Clinical practice**	**Weighted estimates for Europe (respondents from 16 countries with >=10 replies)**			**Other respondents (61 countries)**			
	**N=1281**			**N=449**			
	%*	**95% CI**		**N**	**%**	**95% CI**	
*In my ICU, hand hygiene is done with alcohol hand rub, always, or most of the time*	95	94	97	395	88	85	91
*In my ICU, there are written guidelines for VAP prevention*	65	62	69	282	63	58	67
Guidelines developed locally	33	30	36	162	36	32	41
Guidelines developed nationally	31	28	34	117	26	22	30
*In my ICU, care for intubated patients includes…*							
No ventilatory circuit changes unless specifically indicated	69	66	72	371	83	79	86
Strict hand hygiene using alcohol, especially before managing the airways	83	80	86	364	81	77	85
Systematic daily interruption of sedation and weaning protocol	49	46	53	285	63	59	68
Oral care with chlorhexidine	70	67	73	302	67	63	72
Cuff pressure control at least every 24 hours	83	81	85	347	77	73	81
Head of bed elevation	96	94	97	442	98	97	100

* Absolute numbers are not reported because percentages are weighted estimates.

**Table 4:** VAP prevention: measurements, as reported by ICU doctors

**Measurements**	**Weighted estimates for Europe (respondents from 16 countries with >=10 replies)**			**Other respondents (61 countries)**			
	**N=1281**			**N=449**			
	%*	**95% CI**		**N**	**%**	**95% CI**	
*Measurement of compliance at least once a year*							
Hand hygiene recommendations	57	54	60	265	60	54	64
Systematic daily interruption of sedation and weaning protocol	28	24	33	102	23	19	27
Oral care with chlorhexidine	27	23	30	126	28	24	32
*“In my ICU, there is a written definition of VAP for data collection”*	50	47	54	286	64	59	68
YES- European guidelines	26	23	29	37	8	6	11
YES- CDC guidelines	12	10	15	206	46	41	51
*“In my ICU, we count and record, routinely…” (% saying “yes”)*							
VAP	55	51	58	287	64	59	68
Intubation-days	81	78	84	364	81	74	85
Intubated patients	90	88	92	367	82	78	85
*Please provide, if possible, the following data for your ICU - for part or all 2011 (% providing data)*							
VAP/ 1000 ventilation-days	20	17	22	113	25	21	29
Mean duration of intubation for intubated patients (days)	27	25	30	148	33	29	37
Proportion of intubated patients	38	35	41	178	40	35	44
*“Clinical staff in my ICU is aware of VAP-related measures, and their trends” (% agree strongly / agree)*	53	50	56	298	66	62	71

* Absolute numbers are not reported because percentages are weighted estimates.

Attitudes towards the implementation of a VAP measurement system are presented in Table 5.

**Table 5:** Attitudes towards the implementation of a measurement system of infections in ICUs

	**Weighted estimates for Europe (respondents from 16 countries with >=10 replies)**						**Other respondants (66 countries)**					
	**N=1281**						**N=449**					
	**Agree strongly/ agree**			**Disagree/ disagree strongly**			**Agree strongly/ agree**			**Disagree/ disagree strongly**		
	**%**	**95% CI**	**%**	**95% CI**	**%**	**95% CI**	**%**	**95% CI**				
***To what extent do you agree with the following comments***												
If you cannot measure it, you cannot improve it	83	80	85	11	9	13	84	80	87	11	8	14
Monitoring of VAP related measures stimulates quality improvement	93	91	94	2	1	3	97	94	98	1	0	3
VAP-related measures in my ICU (if any) are reliable	54	51	58	12	10	15	66	61	70	8	6	11
I am willing to implement, or support, a VAP data collection system	84	81	86	4	3	6	92	89	94	1	0	3
Clinical diagnosis of VAP is difficult: this makes measurement systems unreliable	46	43	50	32	29	36	43	38	47	36	32	41
There is a difference between a definition of VAP for reporting, and a diagnosis of VAP for treatment	45	42	49	32	28	35	46	41	50	30	26	35
***Please indicate what actions would facilitate the implementation of a measurement system of infections in ICUs***												
Timely feed-back of data at ICU level	92	90	94	1	1	2	96	93	97	0	0	2
Administrative support	88	86	90	2	1	3	95	92	97	1	0	2
Dedicated software / IT resources	91	89	93	2	1	3	92	89	94	0	0	2
Reliable data	95	93	96	1	0	2	96	94	98	0	0	1

## Discussion

### Key results

This is, to our knowledge, the first international survey assessing VAP prevention practices – (clinical, and measurement) – among ICUs doctors. Participation was large, and almost two thirds of respondents reported the existence of written VAP prevention guidelines in their ICU - pointing out the interest in, and awareness of the problem. If we combine the good clinical practice, AND measuring compliance to this practice at least once a year (a very pragmatic objective), this was reported by 57% (hand hygiene), 29% (daily interruption of sedation) and 26% (oral care with chlorhexidine) of the participants to this survey (European estimates). Interestingly, “head of bed elevation” - a practice ranked very low in the “European care bundle” because it was perceived as difficult to implement - was mentioned by 96% of the respondents; this clinical practice was known by 85% of European nurses participating in a knowledge test about VAP prevention practices [25].

As regards measurement of outcomes, European estimates show that only 54% count and record the number of VAP on a routine basis; and only 20% were able to provide data for their ICU on the main indicator used to monitor VAP - (VAP/1000 intubation-days). In contrast with these low proportions 93% agreed that “monitoring of VAP-related measures stimulates quality improvement” and 84% said they were willing to implement, or support, a VAP data collection system. They expressed some distrust as regards the data (46% agreed with the statement “clinical diagnosis of VAP is difficult; this makes measurement unreliable”), on the other hand, only 50% were aware of a standardized case definition for VAP recording in their ICU; and only 45% understood the difference between a definition of VAP for recording, and a diagnosis of VAP. Overall, 95% of respondents agreed that reliable data would facilitate the implementation of a measurement system.

These European estimates mask large difference between countries. For example oral care with chlorhexidine was reported by 55% (139/251) of the respondents in France, and by 94% (276/293) in Spain. Respondents saying yes to the question “in my ICU, we count and record VAP on a routine basis” were 50% (57/115) in the UK, and 74% (218/293) in Spain. Daily sedation vacation and weaning protocol were reported by 81% (93/115) in the UK, and by 35% (66/187) in Italy (see country-specific data, as Additional file 1).

Results from the 449 respondents not included in the European estimates are surprisingly similar to those of the European estimates.

### Strengths and Limitations

This survey has several limitations. First, we cannot claim that participants represent a random sample of ICU clinicians in Europe nor in their own country. Some categories of ICU doctors are likely to be overrepresented, such as members of ICU national, or international societies. These might be better informed, and apply VAP prevention guidelines more than the average clinician. The list of VAP prevention measures we used as a reference for good practices guidelines [10] could be criticized on several grounds, e.g. it does not include subglottic secretions drainage [26]. Our dissemination strategy obviously worked better in some countries than others. Another limitation is that some questions in the questionnaire apply to the individual physician and others to the ICU (“in my ICU, care for intubated patients includes…) but the online questionnaire did not include questions allowing for the identification of the ICU, in order to preserve the anonymity of the respondents.

Respondents not included in the European estimates represent a very heterogeneous population with no clear geographical basis. We nevertheless considered it worthwhile to pool these results, because together these doctors are responsible for a large number of patients, and these data have identified weaknesses broadly similar to those observed in the European estimates.

Clearly, it cannot be concluded from a doctor’s reporting of a clinical practice in her/his setting, that this practice is used all the time for every patient who needs it: self-reports mainly provide information regarding clinicians’ knowledge of guideline recommendations, but they are subject to bias – overestimation – and should not be used as the sole measure of guideline adherence [27]. Measuring compliance to guidelines at local level once a year appears as an absolute minimum. We did not ask details on the methods used to measure compliance. This is not necessarily easy, for instance detailed guidelines exist for measuring compliance to hand hygiene recommendations; [28] and oral care in ventilated patients is a complex procedure that might require a check list [29].

It was not among the objectives of this survey to collect data on VAP incidence rate in ICUs – there are much better sources for this – e.g. surveillance data for Europe [1]. Rather we wanted to investigate the knowledge doctors had of the rates in their units. However data provided on VAP rates (not shown) are in the expected range as reported in surveillance networks [1], giving some validity to our results.

### Interpretation

The large participation to this survey reflects the interest of the ICU community in the issue of VAP prevention. To the extent that the selection bias, and the reporting bias in our results lead to overestimating VAP prevention practices in ICUs, weaknesses identified appear robust enough as to support targeted interventions for improvement. The priority for improving care of intubated patients is promoting the clinical practices with the lowest reported use (daily sedation vacation, and weaning protocols, oral care with chlorhexidine, and no ventilatory circuit change unless specifically indicated). Improving knowledge of clinical guidelines is far from sufficient to improve practices [25,30] but it is a prerequisite. ICUs doctors overwhelmingly agree that monitoring of VAP-related measures stimulates quality improvement but very few do it, although most are willing to do it. They could be helped to do so by learning how to produce reliable data (standardized case-definitions, methods for measuring compliance) with real-time feed-back at the ICU level; so that clinical staff could monitor their own trends over time. A compromise needs to be found between time-consuming data collection, and usefulness of data. Additional resources (human resources, information technology) might help, but some very simple measures can be implemented with minimal input, e.g. in some ICUs a panel with the number of days since last ICU-acquired infection (including VAP) is displayed on the board and updated daily (personal observations in Scotland, MLL). This study did not consider the issue of surveillance and reporting of outcome indicators at regional or national level, nor the merits (or otherwise) of evaluation of performance and feed-back, based on benchmarking (e.g. comparisons between units). Specific priorities might differ between countries.

## Conclusions

This survey has documented a large potential for improvement in clinical and non-clinical practices aimed at preventing VAP in ICUs. Some results, such as a large agreement of the respondents that data collection is essential – “if you cannot measure it, you cannot improve it” - extend beyond the issue of VAP prevention. Promoting the implementation of guidelines for VAP prevention needs to be done together with promoting the measurement of compliance to these guidelines and measurement of outcomes as a tool for improvement, keeping data collection systems at ICU level as simple as possible: what is important is usefulness, not perfection [31].

## Competing interest

The authors declare that they have no competing interests.

## Authors’ contributions

MLL, MP, AA, MH and UF designed the study. MLL and AI collected and analyzed the data. MLL wrote the first draft of the article. MP, AA, MH, AL, SB, AI and EPH revised the report. All authors saw and approved the final report.

## Supplementary Material

Additional file 1 Detailed country-specific results: number responding, respondents characteristics, reported practices, attitudes. Click here for file

## Data sharing

Data are freely available and have been deposited in the Dryad Digital Repository http://datadryad.org doi:http://10.5061/dryad.q1d7f Further use and exploitation of these data is encouraged.
